# Debonding Failure Analysis of Reinforced Concrete Beams Strengthened with CFRP Plates

**DOI:** 10.3390/polym13162738

**Published:** 2021-08-16

**Authors:** Rendy Thamrin, Zaidir Zaidir, Silvy Desharma

**Affiliations:** Department of Civil Engineering, Faculty of Engineering, Andalas University, Padang 25163, West Sumatera, Indonesia; zaidir2000@yahoo.com (Z.Z.); sydesharma@gmail.com (S.D.)

**Keywords:** debonding load, CFRP plate, RC beams, flexural strengthening, simple statistical analysis, fiber element method

## Abstract

In this study, experimental work was carried out on reinforced concrete (RC) beams strengthened with carbon fiber reinforced polymers (CFRP) plates. This study aims to examine the effect of the reinforcement ratio on the flexural behavior of these beams and propose a new model for predicting the debonding moment. Six RC beams consisting of three control beams and three beams strengthened with CFRP plates were tested. The beams were simply supported and loaded with four-point bending. The test variable was the tensile reinforcement ratio (1%, 1.5%, and 2.5%). Analytical prediction using the fiber element method was also carried out to obtain the complete theoretical response of the beam due to flexural loads. The test results show that the reinforcement ratio affected the bending performance of RC beams with CFRP plates. Following this, the experimental data from 60 beam test results from published literature and this study were analyzed. From these data, it was found that the ratio of tensile reinforcement, the ratio of modulus of elasticity of concrete, the modulus of elasticity of the plate, and plate thickness all affect the value of debonding moment. A parametric study using fiber elements and the two-dimensional finite element method was also carried out to confirm the effect of these variables on debonding failure. These variables were then used to develop an equation to predict the debonding moment of RC beams strengthened with CFRP plates, using simple statistical analysis. This analysis resulted in a simple model for predicting the debonding moment. Then the model is entered into a computer program, and the complete response of the cross-section due to debonding failure can be obtained.

## 1. Introduction

Buildings that experience deterioration in strength, damage, or changes in the design code during their service lifetime need to be strengthened or repaired to meet design code specifications. One way to do this is to use externally bonded Carbon Fiber Reinforced Polymer (CFRP) plates. These have been extensively researched and used to increase the bending capacity of reinforced concrete structures for more than three decades and are reported to have shown promising results [1,2,3,4,5,6,7,8,9,10,11,12,13,14,15,16,17,18,19,20]. This strengthening method is practical and easy to implement and has other advantages such as corrosion resistance, high relative stiffness, and light weight [2,3,6,7].

Experimental studies of externally strengthened RC beams with CFRP plates that several previous researchers have carried out showed premature failure indicated by the loss of the bond between the concrete and the CFRP plate [1,2,3,4,5,6,7,8,9,10,11,12,13,14,15,16,17]. The loss of this bond may or may not result in a separation of the concrete cover layer. Therefore, the capacity of the beam strengthened with CFRP plates is limited to avoid this debonding failure [8]. This limitation means that a large part of the CFRP plate’s capacity to carry the load is unused. As a result, only about 20–30% of the overall capacity of the CFRP plate can be used in the strengthened concrete beam [20].

Previous research has shown that the capacity of a beam strengthened with CFRP plates is influenced by several variables, including the tensile reinforcement ratio [6], the thickness of the CFRP plate [15,18], the modulus of elasticity of CFRP plate [18], and the compressive strength of concrete [18,19]. Nguyen et al. [6] conducted a study on the effect of the reinforcement ratio on the flexural capacity of reinforced concrete beams with CFRP plates, comparing over-reinforced beams and under-reinforced beams. The results showed that the increase in the flexural capacity of over-reinforced beams was smaller than that of under-reinforced beams.

The effect of variations in the CFRP plate layer on reinforced concrete beams was demonstrated in the study by Ahmed et al. [15]. Those studies indicate that the load capacity increases with the number of layers of CFRP plates on the tensile surface of the beam. In a study conducted by Sayed [18], the thickness of the FRP plate (tf) had an effect on the ultimate bond strength of tf to the power of 0.41 (t_f_^0.41^) when the attachment length of the plate was less than the effective attachment length, and tf to the power of 0.32 (t_f_^0.32^) when the attachment length was more significant than the effective attachment length.

The effect of the modulus of elasticity of the FRP plate (Ef) on the ultimate bond strength was studied by Sayed [18]. Sayed found that the effect was Ef to the power of 0.34 when the attachment length was less than the effective attachment length, and Ef to the power of 0.59 when the attachment length was more significant than the effective attachment length. Sayed [18] also found that the ultimate bond load capacity of FRP plates in concrete increased by *fc’* to the power of 0.34 due to the influence of the compressive strength of the concrete (*fc’*). In another study, Mansour [19] showed that the compressive strength of concrete affects the load capacity of reinforced concrete beams strengthened with FRP plates, where the load capacity increases along with the increase in the compressive strength of the concrete.

In previous studies, models for predicting flexural debonding moment in FRP-plated reinforced concrete beams were proposed by Oehlers [21] and Teng and Chen [22]. Oehlers [21] formulated an empirical equation to predict the flexural debonding moment, which is influenced by: the elastic modulus of the concrete, the elastic modulus of the FRP, the cracked second moment of the area of the plated section, the cylinder splitting tensile strength of concrete, and the thickness of the FRP plate. The equation proposed by Teng and Chen [22] was influenced by the theoretical ultimate moment of the unplated section, which is also the upper bound of the flexural debonding moment, the flexural rigidities of the cracked section with and without an FRP plate, the elastic modulus of the FRP, the thickness and width of the FRP plate, the elastic modulus of the concrete, and the width and effective depth of the RC beam.

This present study examines the effect of the reinforcement ratio on the flexural behavior of RC beams with CFRP plates and proposes a model for calculating the debonding moment. In this work, data from previously published studies regarding the flexural strengthening of reinforced concrete beams by externally attaching CFRP plates to the tensile surface were collected, providing a database of 60 experimental beam test results from 17 studies. These were analyzed to achieve the objectives of this study. Cross-sectional analysis based on theoretical moment-curvature determination was also carried out to theoretically predict the flexural capacity of CFRP-plated RC beams. The calculation process was supported by a computer program called Reinforced Concrete Cross Section Analysis (RCCSA) [23,24,25]. The moment-curvature relationship obtained from the analysis can then be used to obtain a complete response of the load-deflection relationship. Then, the proposed debonding moment model is entered into a computer program, and a full response from the cross-section due to debonding failure can be obtained. The analytical results were then compared with those obtained from laboratory testing.

## 2. Experimental Study

**Figure 1:**
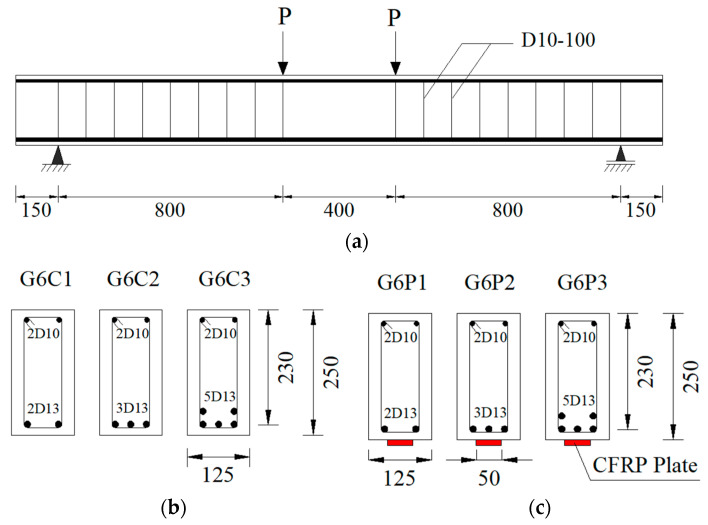
Schematic pictures of the tested beams and their identifications (**a**) beam dimension and loading position, (**b**) cross section of control beams, and (**c**) cross section of plated beams.

The study by Thamrin et al. [1] consisted of six concrete beams, three of which were reinforced with a tensile longitudinal reinforcement ratio of 1%, 1.5%, or 2.5%. The beams tested were classified into control beams (G6C1, G6C2, and G6C3) and beams strengthened with CFRP plates (G6P1, G6P2, and G6P3). The cross-section of each of the beams was 125 × 250 mm, and each had an effective depth of 230 mm. The beams were simply supported and had a length of 2000 mm with an overhang of 150 mm on each side; hence the total size of the beam was 2300 mm, as shown in Figure 1.

The compressive and transverse reinforcement for all beams were 10 mm diameter bars with a yield strength of 355 MPa. The tensile reinforcement used in the test specimens had a 13 mm diameter and a yield strength of 448 MPa. Closed type transverse reinforcement with 100 mm spacing was used on all tested beams to avoid premature collapse due to shear forces. A ready-mix company supplied the fresh concrete, and the compressive strength of the concrete at the age of 28 days was 20 MPa.

**Figure 2:**
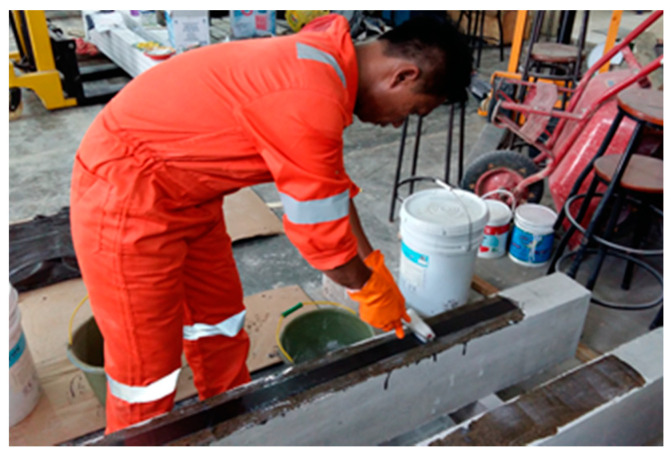
Installing the CFRP plate at the bottom of the beam.

The CFRP plates used were obtained from a roll of Tyfo^®^ UC Composite Laminate Strip laminated by FYFE Co. LLC, USA. The CFRP plates were 1.9 mm thick and 50 mm wide. CFRP plates were glued to the tension face of the strengthened beams with epoxy adhesive by FYFE Co. LLC, as shown in Figure 1c and Figure 2. Tyfo^®^ S Epoxy was applied as the main layer on the prepared concrete substrate and Tyfo^®^ TC Epoxy was applied to a thickness of 2 mm on the substrate before applying the CFRP plate. Installation of CFRP plates on beams was carried out by certified applicators, as shown in Figure 2. The mechanical properties of the CFRP plate used were supplied by the manufacturer. The ultimate tensile strength in primary fiber direction and tensile modulus of the CFRP plates used were 2.51 GPa and 139 GPa, respectively.

**Figure 3:**
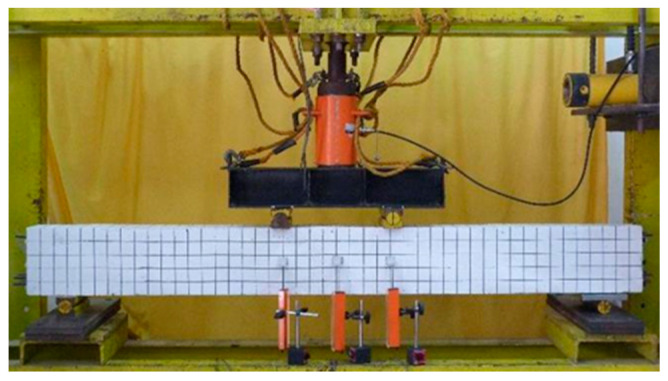
Test setup and equipment used.

Testing of simply supported beams was carried out by applying two concentrated loads 400 mm apart. The load was applied by a 500 kN capacity of a hydraulic actuator. Load cell and linear variable displacement transducers (LVDTs) were used to measure the deflection of the beams. Load cell and LVDT’s were connected to a data acquisition system, and the data was collected on data media. The deflections of the beam located at three positions were recorded continuously. In each test, the load was gradually increased until collapse occurred. Test setup, the position of the load, and LVDT’s on the tested beam are shown in Figure 3. The load cell, LVDT’s and data logger used were products of Tokyo Measuring Instruments Laboratory Co., Ltd., Tokyo, Japan.

## 3. Fiber Element Method

**Figure 4:**
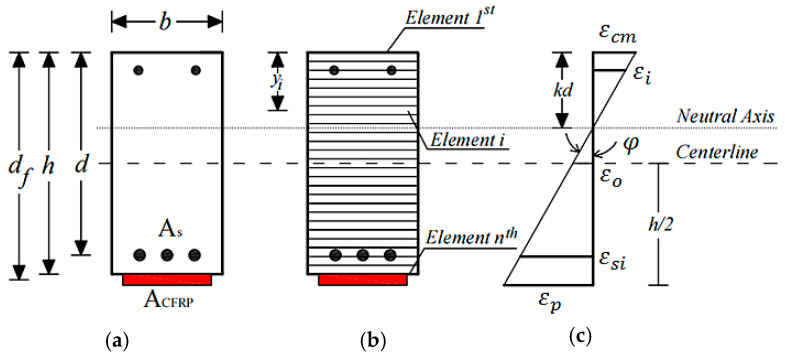
Analytical model using the fiber element method (**a**) reinforced concrete cross section, (**b**) fiber element model, and (**c**) strain distribution.

Flexural analysis of the reinforced concrete cross-section using the fiber element method was carried out to obtain the complete flexural response of the cross-section with CFRP plates due to the applied bending moment. Figure 4 shows the analytical model of the fiber element method. Strain compatibility in this method was applied using the assumption of a perfect bond between concrete and the steel reinforcement as well as between concrete and the CFRP plates. Linear strain was assumed for strain distribution along with the beam depth. The appropriate stress state at each strain position during the calculation process must follow the stress-strain equation of materials. Therefore, the assumed stress-strain relationship for concrete, steel, and the CFRP plate was applied. A bilinear stress-strain model for steel bars and a linear model for the CFRP plate were used. The parabolic stress-strain model for concrete in compression was adopted from the literature [26].

The magnitude of the internal forces was then obtained by using the corresponding strain (*εi*, *εsi*, and *εp*) and stress level at each incremental curvature (*φ*) and the equilibrium of the plated cross-section was determined by an iterative process. Once the equilibrium conditions for the cross-section were fulfilled, the moment at the corresponding curvature was calculated by multiplying the internal forces with the corresponding moment arms (*yi*). Then the calculation process was repeated until the maximum compressive strain of *εcm* = 0.003 was reached.

The first step of this method is performed by dividing the cross-section into a finite number of reinforcement and concrete layers, as illustrated in Figure 4. The steel plate is assumed to be the reinforcement layer in the analytical model, and connections between reinforcement layers and concrete layers are assumed to be perfectly bonded. Hence, the strain distribution along the beam cross-section height can be considered linear, as shown in Figure 4.

The strain, *ε_i_*, in the concrete and reinforcement elements for an assumed value of curvature, *φ*, and the lever arm of each element, *y_i_*, can be calculated as:

(1) $$\varepsilon_{i}=\varepsilon_{o}-\left(\varphi y_{i}\right)$$

The second step is calculating the stresses using a given stress-strain equation for concrete and steel. The stresses, σ*_i_*, acting on each reinforcement layer, concrete element and the steel plate can be determined as:

(2) $$\sigma_{i}=f\left(\varepsilon_{i}\right)$$

The stress-strain equation for concrete in compression applied in this study is adopted from the model proposed by Mander et al. [26]. For concrete in tension, a linear model is used up to the maximum concrete tensile strength without a tension stiffening effect. The stress-strain equation for steel bars and steel plates used in this study is based on a bilinear model.

The third step is calculating the internal forces, *F_i_*, for each of the concrete elements and reinforcement layers with an area, *A_i_*, using:

(3) $$F_{i}=A_{i}\sigma_{i}$$

The fourth step is checking whether the equilibrium of internal forces is satisfied. An iterative procedure is required to obtain the value of axial strain, *ε_o_*, which fulfills the equilibrium condition of the internal forces.

The fifth step is calculating the internal moment, *M*, in the cross-section as:

(4) $$M=\Sigma F_{i}y_{i}$$

The last step is calculating the load, *P*, and deflection, *δ*, values by using the appropriate moment and curvature distribution with each incremental step along the length, *L*, of the beam, which is calculated as:

(5) $$\delta =\int_{0}^{\frac{L}{2}}x\varphi dx$$

**Figure 5:**
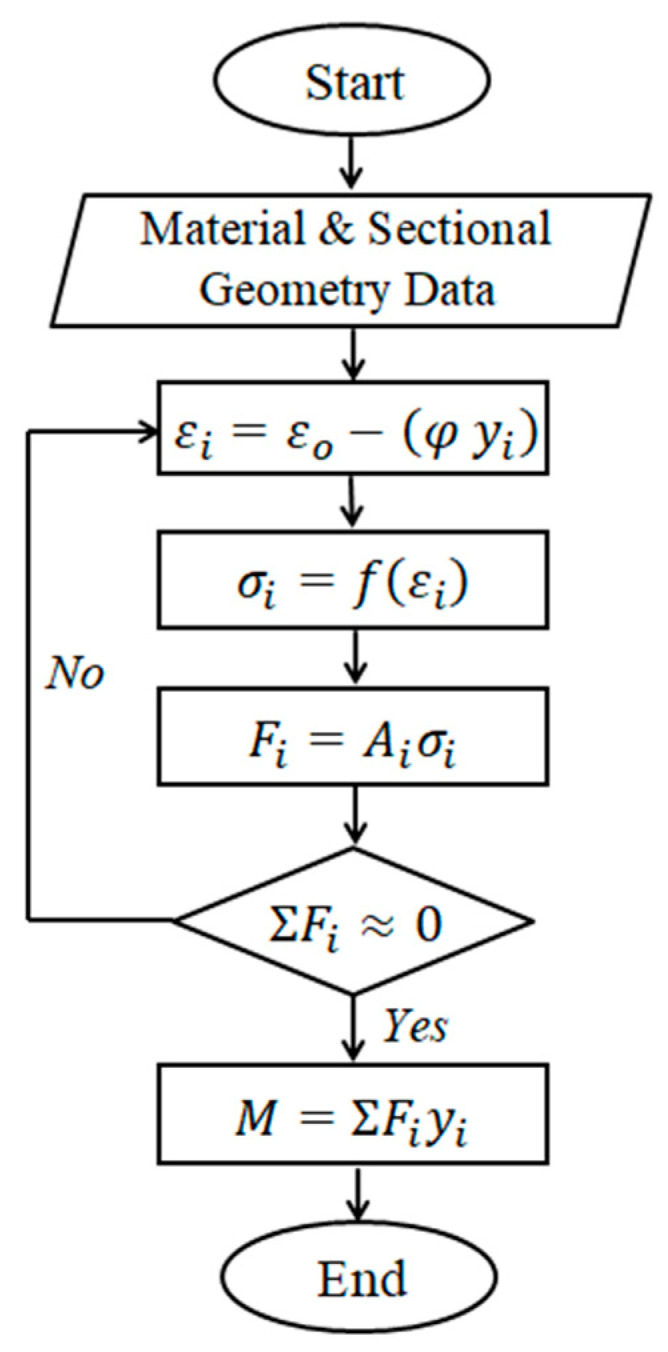
The algorithm of the computation procedure.

The complete details of the computation procedure can be found in the literature [24,25]. The algorithm of the computation procedure is illustrated in Figure 5. A computer program based on the formula above was developed and used to facilitate this process [23,24,25].

## 4. Finite Element Method

**Figure 6:**
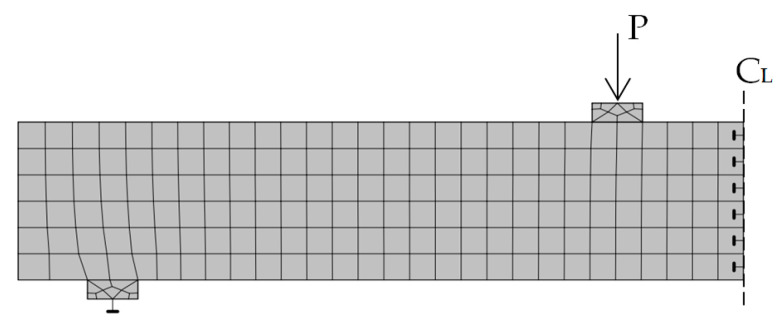
Finite element model of beam specimen using ATENA 2D.

The finite element method was used in this study to analyze the strain behavior of beams in the shear span zone and the load-deflection curves. The 2D ATENA software was used to accomplish this purpose. ATENA is finite element-based software specially designed for reinforced concrete analysis, and a complete explanation of this software can be seen in the literature [27]. The beam model analyzed with ATENA software will be discussed in more detail later. The beam is modeled with a 40 mm quadrilateral element, as shown in Figure 6. Only half of the beam length was used in the finite element model because of the symmetry of the beam’s two-point loads position and geometry. The axes of symmetry in the middle of the beam was simulated by boundary conditions with constraints on horizontal displacement. As in the laboratory test, the acting forces and supports are applied through the steel plate to avoid stress concentrations to the concrete. The load applied to the beam was a load case with prescribed deformations type and the displacement given was 0.1 mm downward.

**Figure 7:**
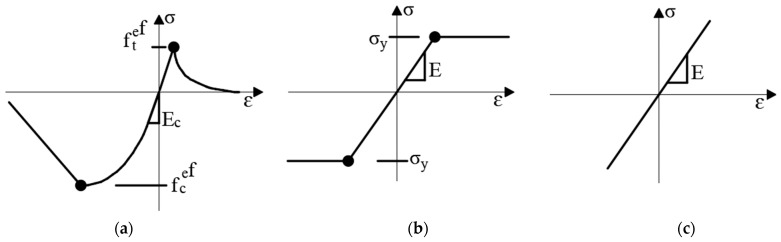
Material stress-strain models used in finite element analysis (**a**) concrete, (**b**) steel reinforcement, and (**c**) CFRP plate.

The plates were assumed to be perfectly bonded to the concrete. Stress-strain models for concrete, reinforcing steel, steel plates, and CFRP plates used the material models available in the ATENA software. The SBETA material model was used for concrete and the bilinear model was used for the steel reinforcement. The loading and support steel plates were modeled as an elastic material, while the CFRP plate was modeled as reinforcement with elastic material. Stress-strain models used in the finite element analysis are shown in Figure 7.

## 5. Results and Discussion

### 5.1. Experimental Results

The results of the experimental study [1] are presented in the form of load-deflection curves and crack patterns. The first crack in the control beam occurred at an average load of 3.3 kN, while for the beam with CFRP plates, the first crack occurred at an average load of 9.4 kN. Based on the test result, all control beams (G6C1, G6C2, and G6C3) and beam strengthened with the CFRP plate (G6P3) failed in flexure as indicated by concrete crushing in the top of the compression zone after the yielding of the tensile reinforcement. Two of the beams that were strengthened with CFRP plates (G6P1 and G6P2) failed prematurely due to the debonding of the CFRP plates indicated by concrete cover separation starting at a plate end and then propagating along the CFRP plate interface toward the middle of the beam.

**Figure 8:**
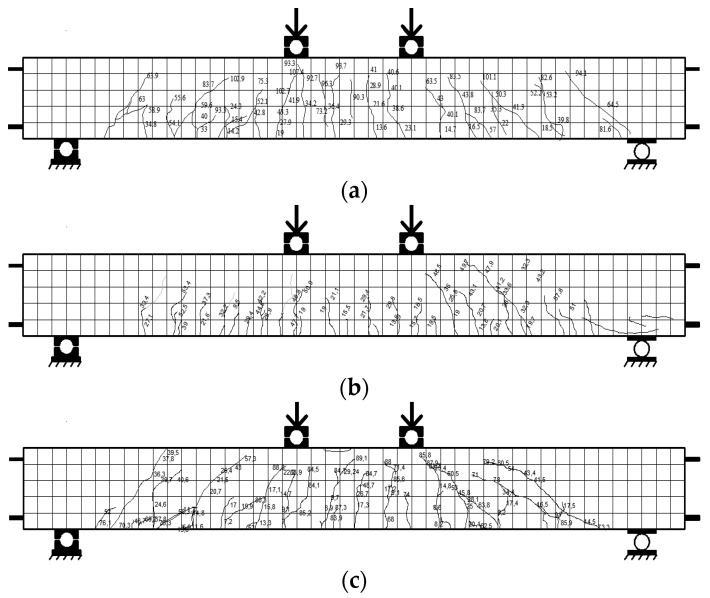

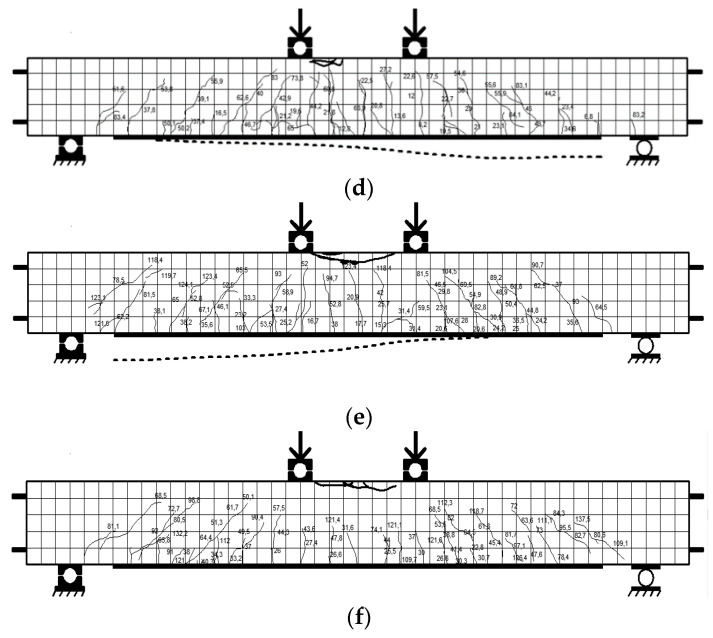
Crack patterns of the beams after the test (**a**) G6C1, (**b**) G6C2, (**c**) G6C3, (**d**) G6P1, (**e**) G6P2, and (**f**) G6P3.

Figure 8 shows the crack pattern of the test beam. The growth of flexural cracks on the stress side of the beam is followed by shear cracks that spread in the shear span zone. In beams with CFRP plate reinforcement, the cracking loads were higher than those in the control beams, due to the contribution of CFRP plates.

The angle of the shear crack was higher in beams with smaller tensile reinforcement ratios, as shown in Figure 8. In reinforced concrete beams with a lower tensile reinforcement ratio, bending behavior was more dominant, while in beams with a higher tensile reinforcement ratio, the shear force increased as flexural capacity increased.

**Figure 9:**
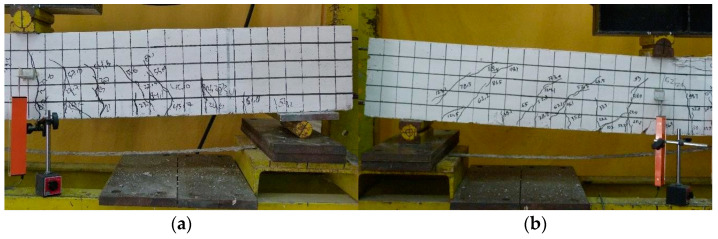
Debonding zone of beams with CFRP plates (**a**) G6P1, and (**b**) G6P2.

As shown in Figure 8, all beams showed a crushing of concrete in the compression zone. This is because, in experimental studies, if debonding failure occurred (for G6P1 and G6P2 beams), the load was continuously applied until the beam reached concrete crushing in the top of the compression zone. The debonding failure between the concrete and the CFRP plate occurred suddenly without any indication of delamination. The failure load occurred at the load levels of 48.9 and 63.1 kN for beams G6P1 and G6P2, respectively. The location of delamination between the concrete and the CFRP plate on the G6P1 and G6P2 beams is shown in Figure 9.

**Figure 10:**
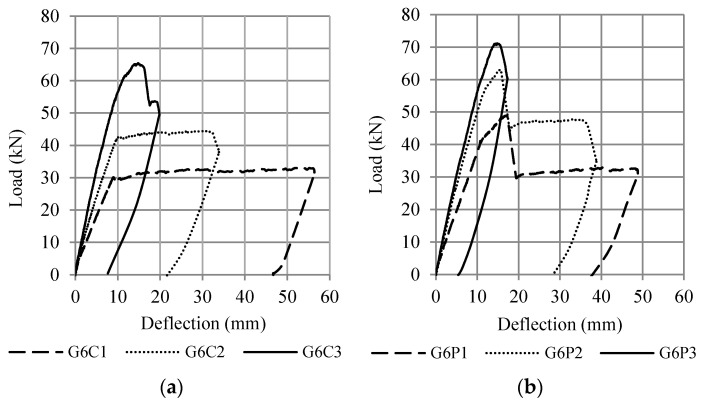
Load-deflection curve from the tested beams (**a**) control beams, and (**b**) beams with CFRP plates.

Figure 10 shows the flexural capacity of the test beam. It can be seen that an increase in the tensile strength ratio causes an increase in flexural capacity but also causes a decrease in beam ductility. A sudden drop in the load-deflection curve occurs due to delamination of the CFRP plate, as shown in Figure 10b. After delaminating the CFRP plate, the load immediately drops to the same load position as the control beam, as shown in Figure 10a. This phenomenon indicates that on the plated beams, the delamination of CFRP on the strengthened beam causes the beam to return to its unplated capacity after the influence of the CFRP plate was removed.

**Figure 11:**
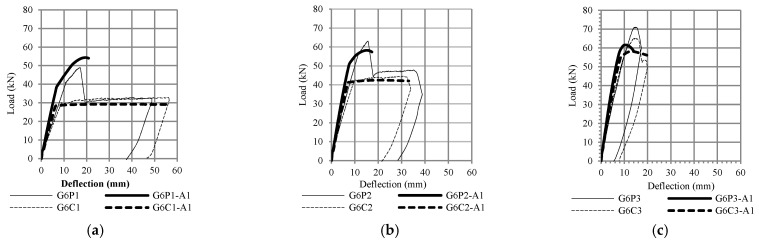
Comparison between test results and the fiber element method (RCCSA) (**a**) reinforcement ratio 1%, (**b**) reinforcement ratio 1.5%, and (**c**) reinforcement ratio 2.5%.

**Figure 12:**
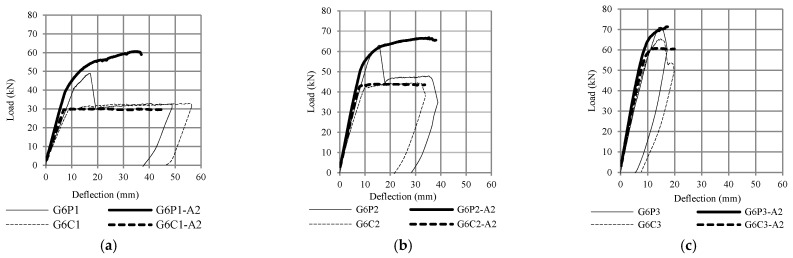
Comparison between test results and the finite element method (ATENA 2D) (**a**) reinforcement ratio 1%, (**b**) reinforcement ratio 1.5%, and (**c**) reinforcement ratio 2.5%.

The flexural capacity of reinforced concrete beams with CFRP plates was 10% to 50% higher than the control beams, depending on the value of the reinforcement ratio, as shown in Figure 11 and Figure 12. Figure 11 compares the results of fiber element method (G6P1-A1, G6P2-A1, G6P3-A1) with experimental results (G6P1, G6P2, G6P3) and Figure 12 compares the results of finite element method (G6P1-A2, G6P2-A2, G6P3-A2) with experimental results. The comparison shows that the fiber element method can predict the flexural response of the test beam with reasonable accuracy. The analysis confirmed that the flexural capacity of beams with CFRP plates is 8 to 55% higher than beams without plates depending on the value of the longitudinal reinforcement ratio. These figures also confirm that debonding failure occurred after yielding of the tensile reinforcement (G6P1 and G6P2).

### 5.2. Parametric Study

**Figure 13:**
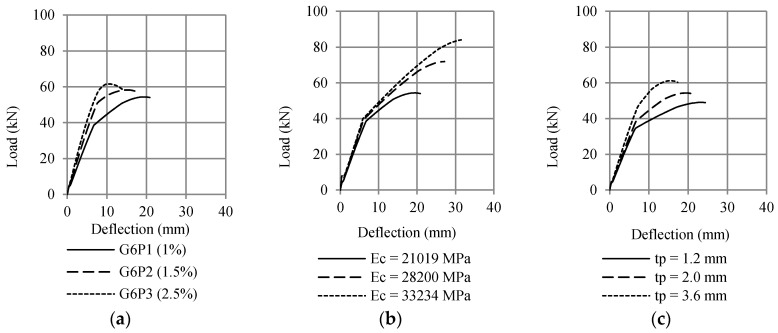
Effect of (**a**) ratio of tensile reinforcement, (**b**) the elastic modulus of the concrete, and (**c**) plate thickness on the flexural capacity using RCCSA.

Moreover, the fiber element method was applied to estimate the effect of tensile reinforcement ratio, modulus elasticity of the concrete, and plate thickness on the flexural capacity of RC beam strengthened with CFRP plate. The results of this analysis are shown in Figure 13. It is shown that all three variables increase flexural capacity.

**Figure 14:**
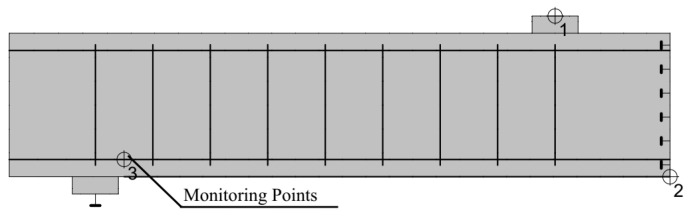
Position of monitoring points in the finite element model.

To examine the effect of the ratio of tensile reinforcement, the elastic modulus of the concrete, and plate thickness on the strain of the concrete in the shear span zone using two-dimensional finite element analysis, ATENA 2D software was used in this study. Figure 14 shows the three monitoring points applied on the finite element model; the first one is to monitor the load, the second is to monitor the deflection at the middle of the beam, and the third one is to monitor the strain on the concrete parallel to the tensile reinforcement and above the end of the CFRP plate.

**Figure 15:**
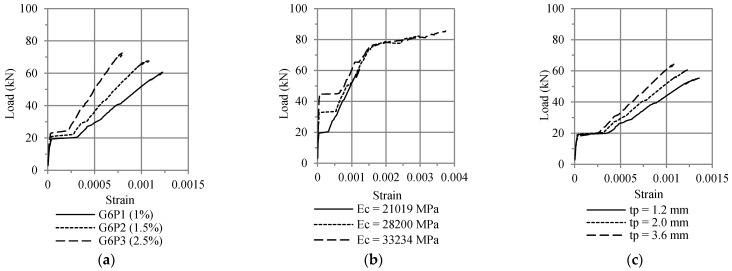
Effect of (**a**) ratio of tensile reinforcement, (**b**) the elastic modulus of the concrete, and (**c**) plate thickness on the strain of tensile reinforcement using ATENA.

The results of the analysis are shown in Figure 15. As the ratio of tensile reinforcement, modulus elasticity of the concrete, and plate thickness increase, the strain at the monitoring positions also increased.

Based on the results of the parametric study above, it was found that the three variables evaluated were the main variables in the debonding failure analysis. These three variables were then used in the model for predicting the debonding moment.

### 5.3. Debonding Failure Analysis

Many empirical equations for flexural debonding moments have been suggested in the literature. Two of them, listed in Table 1, were used in this study. It can be seen in Table 1 that both the equations listed take into account the thickness of the FRP plate, the modulus of elasticity of concrete, and the modulus of elasticity of the plate. The equations use different constant values because of mathematical functions used to model the observed data and the statistical approach used to analyze the data.

In this study, the author modeled the debonding moment using basic equations from bending theory. The equation for calculating the crack moment (*M_cr_)* in a square cross-section of concrete, as shown in Equation (8), was used.

(8) $$M_{cr}=\frac{f_{t}I}{y}$$

where *f_t_* is the tensile strength of concrete, *I* is the moment of inertia, and *y* is the distance from the centroidal axis of the beam cross-section.

Assuming *y* = *h*/2 and a moment of inertia *I* = (1/12) *bh*^3^, we modified Equation (9) as;

(9) $$M_{db}=\frac{1}{6}f_{t}b_{w}h^{2}$$

**Figure 16:**
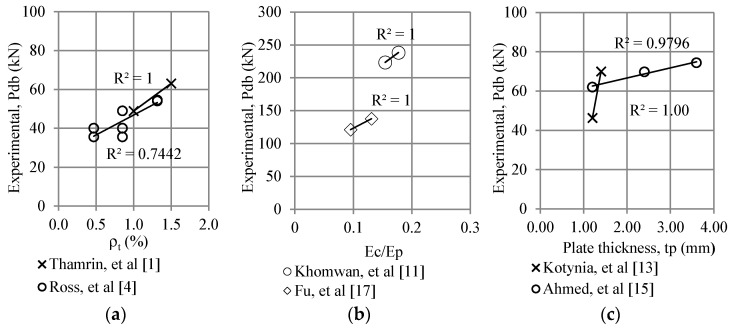
Effect of (**a**) ratio of tensile reinforcement, (**b**) *E_c_*/*E_p_*, and (**c**) the plate thickness on the debonding loads.

The experimental test results of the study by Thamrin et al. [1] and Ross et al. [4] are plotted in Figure 16a to examine the effect of the tensile reinforcement ratio on the debonding load. Data from the experimental study by Khomwan et al. [11] and Fu et al. [17] were plotted in Figure 16b to obtain the effect of the ratio of the modulus of elasticity of concrete (*E_c_*) with that of the plate (*E_p_*) on the debonding load. Furthermore, Figure 16c plots the effect of the CFRP plate thickness on the debonding load using the data from the study by Kotynia et al. [13] and Ahmed et al. [15]. These plot results show that the longitudinal tensile reinforcement ratio, *E_c_*/*E_p_*, and plate thickness ratio significantly affect the debonding load.

Therefore, the three variables that affect the debonding moment applied in the model proposed in this study are: (1) the effect of the longitudinal tensile reinforcement ratio is expressed as α, (2) the ratio of *E_c_*/*E_p_* is expressed as *β* and (3) the effect of plate thickness as *t_p_*. The effect of these three variables is shown in Equation (10).

(10) $$M_{db}=\frac{1}{6}\alpha \beta t_{p}b_{w}h^{2}f_{t}$$

Simple statistical analysis was performed to determine the value of the constants ω and to fit the test data for the debonding moment equation, as expressed in Equation (11).

(11) $$M_{db}=\omega \alpha \beta t_{p}b_{w}h^{2}f_{t}$$

where: ω is a constant for the debonding moment equation and is expressed in a unit of 1/mm, $\alpha =100\rho_{t}$, *ρ_t_* is the ratio of tensile reinforcement, β = *E_c_*/*E_p_*, *E_c_* is the modulus of elasticity of concrete, *E_p_* is the modulus of elasticity of the plate, *t_p_* is the plate thickness, *b_w_* is the width of the RC beam, *h* is the height of the rectangular concrete member, and *f_t_* is the splitting tensile strength; if the splitting tensile strength is not determined from tests, then the value of the concrete tensile strength can be calculated as *f_t_* = *f_c_’*/10.

To assess the accuracy of the proposed model, we used the test results from this study, selected additional data from the literature [1,2,3,4,5,6,7,8,9,10,11,12,13,14,15,16,17], and we summarized the data in a database of 60 existing RC beam tests. The selected beams were simply supported, rectangular or square in cross-section, and retrofitted with CFRP plates on their soffits. The beams in the database list failed either due to flexural failure or debonding. The database includes a wide variety of beam configurations. The beam spans vary from 0.94 to 6.5 m. The beam section aspect ratios, width over depth, range from 0.36 to 3.00. The concrete strengths reported ranged from 17.4 to 80 MPa. The CFRP plate thickness varied from 0.45 to 3.6 mm. The variation of the tensile reinforcement ratio ranged from 0.39% to 10.17%. The assessment of the proposed debonding load formula (with ω = 6) was carried out on data points of adopted data from RC beams strengthened with CFRP plates. The results can be seen in Table 2.

**Figure 17:**
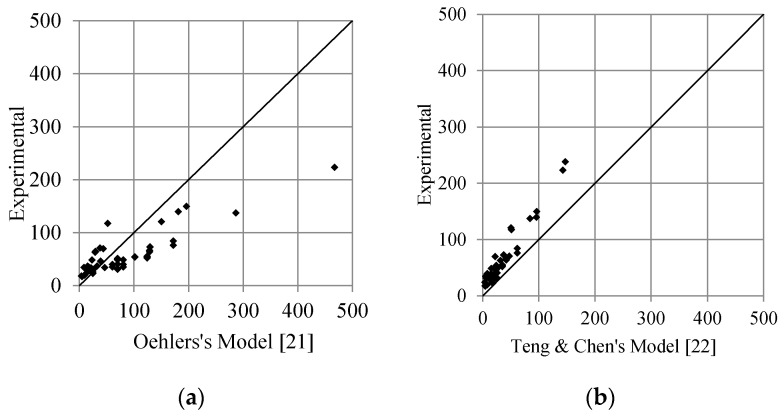
Comparison of the literature models of the debonding load (**a**) Oehler’s Model [21], and (**b**) Teng & Chen’s Model [22], with experimental results.

**Figure 18:**
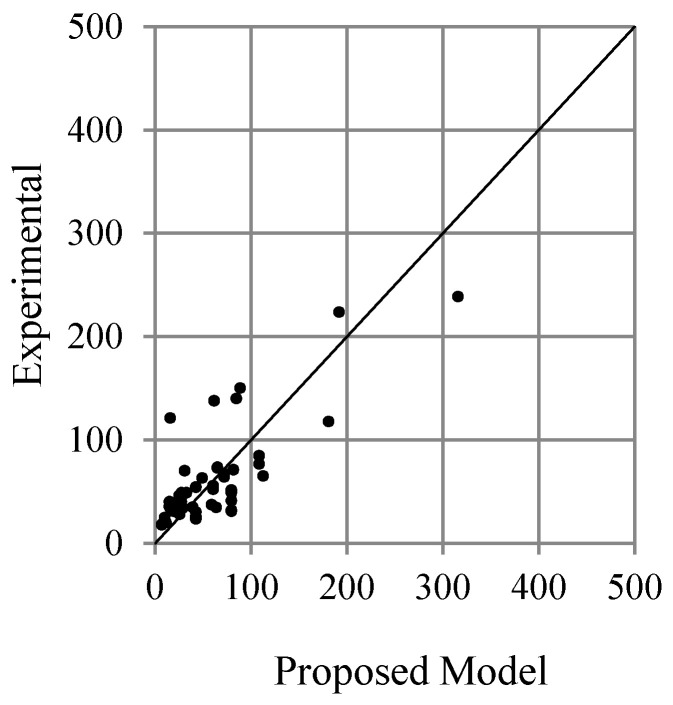
Comparison of the debonding load between the proposed model (ω = 6) and the experimental results.

Comparison of literature models of the debonding load (Oehlers [21] and Teng and Chen [22]) with adopted 60 experimental test specimens are shown in Figure 17. It can be seen that both equations predict the debonding load conservatively, although some data are not conservative. Figure 18 compares the collected data results with the proposed model. The graph shows that the proposed model predicts the experimental results as well as the other models.

### 5.4. Implementation of the Proposed Model on the RCCSA Software

To check the validity of the proposed model, Equation (11) was implemented in the RCCSA software. The value of the debonding moment can be calculated after all of the variables in Equation (11) are entered into the data input block. For each step of incremental curvature, the obtained moment value is checked. The axial stiffness of the CFRP plate is removed when the moment value reaches the debonding moment value obtained from Equation (11).

**Figure 19:**
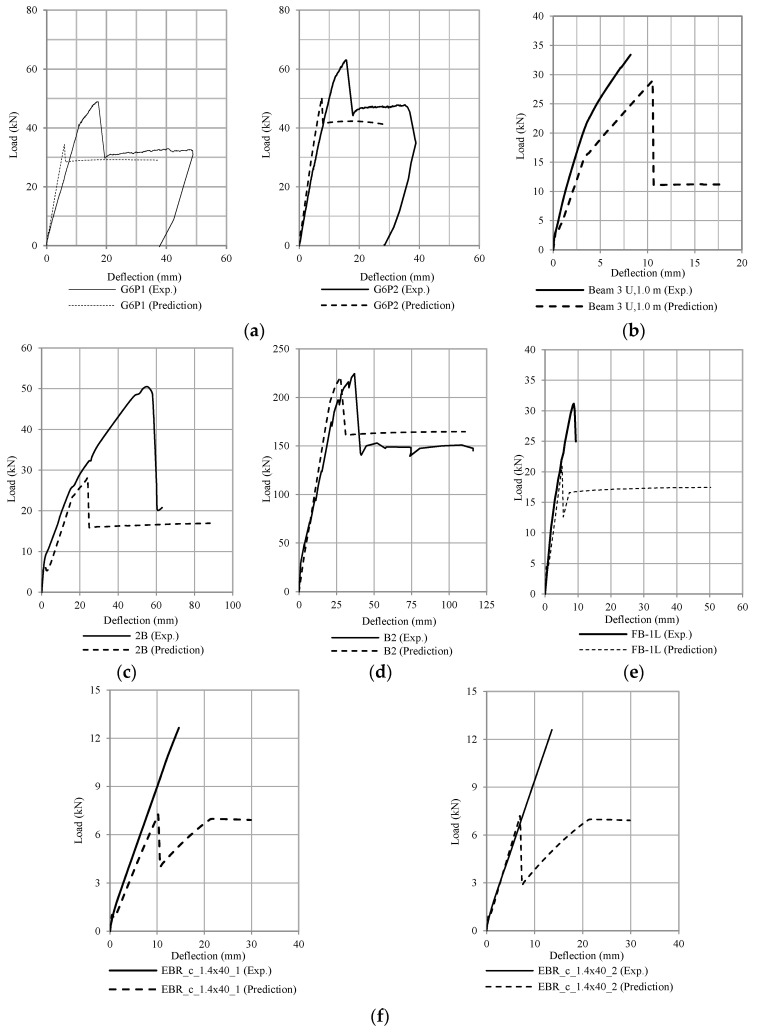
Comparison of the debonding load between the experimental results and the prediction (Type 1 debonding failure occurs before the experimental debonding load and after the yielding of tensile reinforcement) (**a**) Thamrin et al. [1], (**b**) Garden & Hollaway [2], (**c**) Ross et al. [4], (**d**) Khomwan et al. [11], (**e**) Ahmed et al. [15], and (**f**) Bilotta et al. [16].

**Figure 20:**
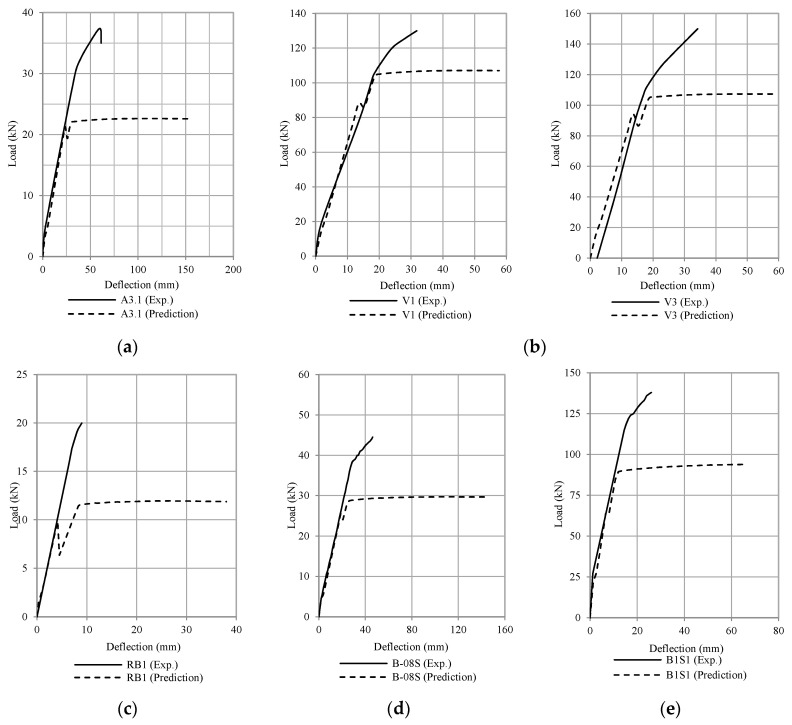
Comparison of the debonding load between the experimental results and the prediction (Type 2. debonding failure occurs before the yielding of tensile reinforcement) (**a**) Spadea et al. [3], (**b**) Shehata et al. [5], (**c**) Benjeddou et al. [12], (**d**) Kotynia et al. [13], and (**e**) Fu et al. [17].

**Figure 21:**
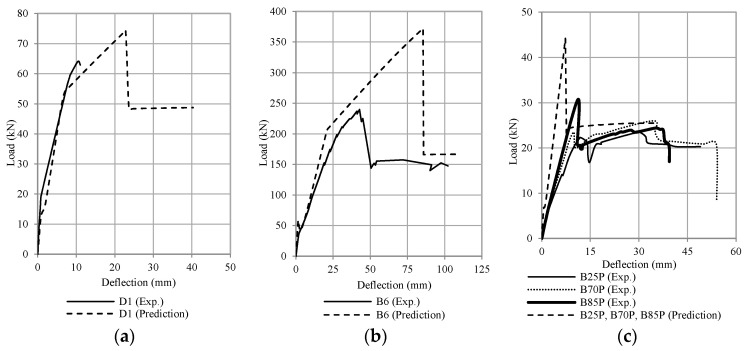

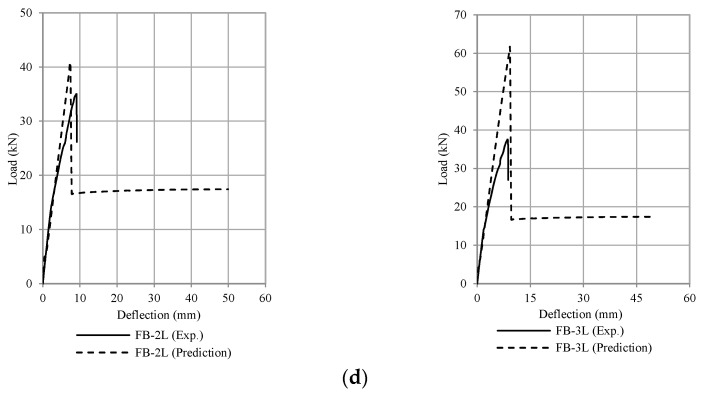
Comparison of the debonding load between the experimental results and the prediction (Type 3. debonding failure value is higher than the experimental debonding load) (**a**) Brena et al. [8], (**b**) Khomwan et al. [11], (**c**) Al-Tamimi et al. [14], and (**d**) Ahmed et al. [15].

**Figure 22:**
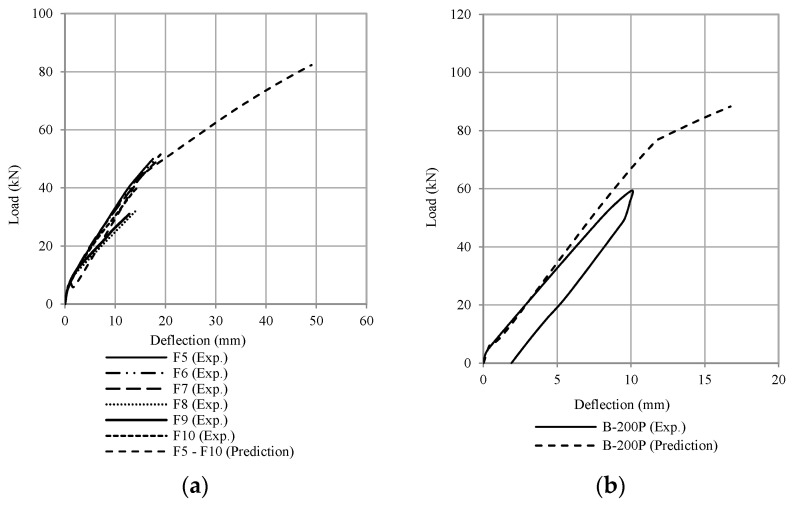
Comparison of the debonding load between the experimental results and the prediction (Type 4. the value of the calculated debonding moment is higher than the ultimate nominal moment of the cross-section) (**a**) Fanning and Kelly [7], and (**b**) Pimanmas et al. [10].

Figure 19, Figure 20, Figure 21 and Figure 22 compare the calculation results (using ω = 6) with some experimental data adopted from the literature [1,2,3,4,5,6,7,8,9,10,11,12,13,14,15,16,17]. The occurrence of debonding failure results in a sudden drop of load on the load-deflection curve, as shown in Figure 19, Figure 20 and Figure 21. From these plots, based on the prediction results using Equation (11), there are four types of results. The four types are: (1) debonding failure occurs before the experimental debonding load and after yielding of the tensile reinforcement (see Figure 19), (2) debonding failure occurs before yielding of the tensile reinforcement (see Figure 20), (3) debonding failure value is higher than experimental debonding load (see Figure 21), and (4) the value of the calculated debonding moment is higher than the ultimate nominal moment of the cross-section (debonding failure does not occur, see Figure 22).

The comparison between analytical prediction and experimental data show a considerable variation due to several factors, including the bond behavior between the FRP plates and concrete, which is not the same in every case reviewed in this study; this is not the scope included in this study. The constant ω was then adjusted to obtain the value that represents all data. Figure 19 and Figure 20 show conservative predictions using the value of ω = 6. However, this value does not give a conservative prediction for the other experimental data, as shown in Figure 21 and Figure 22. From the comparison results shown above, it can be concluded that the value of ω = 6 will give a less conservative value. Therefore, the author recommends a value between 5 and 6 to provide a conservative value. A value of ω lower than 5 will result in a too conservative value, and most of the capacity of the CFRP plate to carry the load is unused. On the other hand, a value of ω greater than 6 will result in a high debonding moment value, which means not safe.

## 6. Conclusions

Bending capacity and debonding load of six reinforced concrete beams with and without CFRP plates were measured and the results were combined with an extensive dataset drawn from the existing literature. These data were used to construct a predictive mathematical model for debonding moments. Results from this model were then compared with the bending capacity of the beam with the different tensile reinforcement ratios from the experimental test results and previously proposed models. The following conclusions are drawn.

Reinforced concrete beams strengthened with CFRP plate have a 10% to 50% higher flexural capacity than unplated beams depending on the value of the tensile reinforcement ratio.

Failures in reinforced concrete beams with small reinforcement ratios (1% and 1.5%) are debonding failures due to the delamination of CFRP plates. In reinforced concrete beams with a reinforcement ratio of 2.5%, flexural failure occurs without delamination on the CFRP plate. These results indicate that an increase in the tensile reinforcement ratio causes a reduction in the stress on the CFRP plate.

The ratio of tensile reinforcement, plate thickness, the ratio of modulus of elasticity of concrete, and the modulus of elasticity of the plate all affect the debonding load of reinforced concrete beam strengthened with the CFRP plate at the bottom of the beam.

The fiber element method predicts the response of reinforced concrete sections with CFRP plates with a high degree of accuracy. The debonding load calculated using the proposed model compares conservatively to the experimental value.

Due to safety considerations and optimization of the capacity of the CFRP plate to carry the load, the value of ω in Equation (11) is proposed to be between 5 and 6.

## Figures and Tables

**Table 1:** Empirical equations for debonding moment from the literature.

Literature	Empirical Equations for Debonding Moment	
Oehlers [21]	$M_{db,f}=\frac{E_{c}\;I_{tr,c}\;f_{ct}}{0.901\;E_{frp}\;t_{frp}}$	Equation (6)
Teng & Chen [22]	$M_{db,f}=\frac{0.488\;M_{u,0}}{{\left(\alpha_{flex}\alpha_{axial}\alpha_{w}\right)}^{1/9}}$	Equation (7)

where: $\alpha_{flex}=\frac{\left[{\left(EI\right)}_{c,frp}-{\left(EI\right)}_{c,0}\right]}{{\left(EI\right)}_{c,0}}$, $\alpha_{axial}=\frac{E_{frp}\;t_{frp}}{E_{c}\;d}$, $\alpha_{w}=\frac{b_{c}}{b_{frp}}$, *M_db,f_* means flexural debonding moment, *E_c_* and *E_frp_* are the elastic modulus of the concrete and the elastic modulus of the FRP, respectively, *I_tr,c_* is the cracked second moment of the area of the plated section transformed to concrete, *f_ct_* is the cylinder splitting tensile strength of concrete, *b_frp_* and *t_frp_* are the width and thickness of the FRP plate, respectively, and *M*_*u,*0_ is the theoretical ultimate moment of the unplated section, which is also the upper bound of the flexural debonding moment *M_db,f_*. *α_flex_*, *α_axial_* and *α_w_* are three dimensionless variables, (*EI*)*_c,frp_* and (*EI*)_*c,*0_ are the flexural rigidities of the cracked section with and without an FRP plate, respectively, and *b_c_* and *d* are the widths and effective depth of the RC beam, respectively.

**Table 2:** Test results and theoretical values of the debonding load.

Specimen	*fc’*	*ft*	*E_c_*	*b_w_*	*h*	*a*	*ρ_t_*	*t_p_*	*E_p_*	Oehlers [21]	Teng&Chen [22]	Proposed Model	P_exp_
	(MPa)	(MPa)	(GPa)	(mm)	(mm)	(mm)		(mm)	(GPa)	(kN)	(kN)	(kN)	(kN)
Thamrin et al. [1]													
G6P1	20	2.0	21	125	250	800	0.009	1.90	139	22.77	21.27	32.71	48.90
G6P2	20	2.0	21	125	250	800	0.014	1.90	139	28.35	31.19	49.06	63.10
G6P3	20	2.0	21	125	250	800	0.023	1.90	139	37.35	47.15	81.77	71.15
Garden & Hollaway [2]													
Beam 1 U,1.0 m	44.8	4.5	31	100	100	300	0.01	0.82	110	15.35	5.96	21.22	36.50
Beam 2 U,1.0 m	44.8	4.5	31	100	100	300	0.01	0.82	110	15.35	5.96	21.22	32.00
Beam 3 U,1.0 m	44.8	4.5	31	100	100	220	0.01	0.82	110	20.93	8.12	28.94	34.00
Beam 4 U,1.0 m	44.8	4.5	31	100	100	100	0.01	0.82	110	46.04	17.87	63.66	34.50
Beam 5 U,1.0 m	44.8	4.5	31	100	100	100	0.01	0.82	110	46.04	17.87	63.66	34.60
Spadea et al. [3]													
A3.1	24.9	2.5	23	140	300	1800	0.011	1.20	152	32.22	18.47	21.14	37.40
Ross et al. [4]													
1B	54.8	5.5	35	200	200	914	0.0047	0.45	138	60.01	7.92	15.19	40.05
1C	54.8	5.5	35	200	200	914	0.0047	0.45	138	60.01	7.92	15.19	35.60
2B	54.8	5.5	35	200	200	914	0.0085	0.45	138	80.06	15.10	27.57	48.95
2C	54.8	5.5	35	200	200	914	0.0085	0.45	138	80.06	15.10	27.57	35.60
2D	54.8	5.5	35	200	200	914	0.0085	0.45	138	80.06	15.10	27.57	40.05
3B	54.8	5.5	35	200	200	914	0.0132	0.45	138	101.33	23.96	42.58	54.52
3C	54.8	5.5	35	200	200	914	0.0132	0.45	138	101.33	23.96	42.58	54.07
3D	54.8	5.5	35	200	200	914	0.0132	0.45	138	101.33	23.96	42.58	54.29
4B	54.8	5.5	35	200	200	914	0.0187	0.45	138	123.76	34.60	60.68	53.82
4C	54.8	5.5	35	200	200	914	0.0187	0.45	138	123.76	34.60	60.68	52.29
4D	54.8	5.5	35	200	200	914	0.0187	0.45	138	123.76	34.60	60.68	55.63
5B	54.8	5.5	35	200	200	914	0.0201	0.45	138	128.85	37.19	65.15	73.43
5C	54.8	5.5	35	200	200	914	0.0201	0.45	138	128.85	37.19	65.15	73.43
5D	54.8	5.5	35	200	200	914	0.0201	0.45	138	128.85	37.19	65.15	72.76
6B	54.8	5.5	35	200	200	914	0.0335	0.45	138	171.54	61.32	108.47	84.55
6C	54.8	5.5	35	200	200	914	0.0335	0.45	138	171.54	61.32	108.47	76.55
Shehata et al. [5]													
V1	33.3	3.3	27	150	450	1350	0.010	1.20	165	180.83	95.39	84.87	140.00
V3	34.3	3.4	28	150	450	1350	0.010	1.20	165	195.75	96.01	88.73	150.00
Nguyen et al. [6]													
A950	26.6	2.7	24	120	150	440	0.016	1.20	181	15.61	14.34	25.80	28.10
A1100	26.6	2.7	24	120	150	440	0.016	1.20	181	15.61	14.34	25.80	28.65
A1150	26.6	2.7	24	120	150	440	0.016	1.20	181	15.61	14.34	25.80	29.45
B1	37.0	3.7	29	120	150	440	0.004	1.20	181	12.73	3.53	10.14	24.60
B2	37.0	3.7	29	120	150	440	0.044	1.20	181	28.95	41.41	112.69	65.05
C5	20.8	2.1	21	120	150	440	0.014	1.20	181	16.67	17.41	15.29	35.50
C10	20.8	2.1	21	120	150	440	0.015	1.20	181	15.74	16.41	15.86	34.00
C20	20.8	2.1	21	120	150	440	0.016	1.20	181	14.02	14.47	17.13	31.50
Fanning & Kelly [7]													
F5	80	5.0	39	155	240	1100	0.011	1.20	155	69.59	25.51	79.64	50.00
F6	80	5.0	39	155	240	1100	0.011	1.20	155	69.59	25.51	79.64	51.50
F7	80	5.0	39	155	240	1100	0.011	1.20	155	69.59	25.51	79.64	48.75
F8	80	5.0	39	155	240	1100	0.011	1.20	155	69.59	25.51	79.64	32.00
F9	80	5.0	39	155	240	1100	0.011	1.20	155	69.59	25.51	79.64	31.00
F10	80	5.0	39	155	240	1100	0.011	1.20	155	69.59	25.51	79.64	41.00
Breña et al. [8]													
D1	37.2	3.7	29	203	406	1220	0.005	1.19	155	128.10	41.78	72.10	64.05
D2	37.2	3.7	29	203	406	1220	0.005	1.19	155	128.10	41.78	72.10	66.95
Breña et al. [9]													
A6-I	47.7	4.8	33	100	100	330	0.008	1.19	155	7.98	5.14	17.44	34.80
Pimanmas et al. [10]													
B-200P	44	4.4	31	120	220	700	0.033	1.20	150	51.45	51.12	180.95	117.79
Khomwan et al. [11]													
B2	37	3.7	25	350	700	2500	0.006	1.40	165	467.07	142.64	191.97	223.50
B6	53	5.3	29	350	700	2500	0.006	1.40	165	574.57	147.06	315.70	238.50
Benjeddou et al. [12]													
RB1	21	1.86	30	120	150	600	0.010	1.20	165	9.20	7.91	11.47	20.06
Kotynia et al. [13]													
B-08 S	32.3	2.8	27	150	300	1400	0.008	1.20	172	38.35	23.28	25.28	46.30
B-08 M	37.3	3.5	29	150	300	1400	0.008	1.40	220	43.51	21.83	30.97	70.00
Al-Tamimi et al. [14]													
B85P	54	5.4	35	110	180	562	0.009	1.40	215	24.21	16.50	42.59	30.35
B25P	54	5.4	35	110	180	562	0.009	1.40	215	24.21	16.50	42.59	25.97
B70P	54	5.4	35	110	180	562	0.009	1.40	215	24.21	16.50	42.59	23.54
Ahmed et al. [15]													
FB-1L	36	3.0	29	150	200	700	0.006	1.20	165	24.85	11.81	19.66	31.00
FB-2L	36	3.0	29	150	200	700	0.006	2.40	165	17.30	10.18	39.32	34.88
FB-3L	36	3.0	29	150	200	700	0.006	3.60	165	14.52	9.35	58.98	37.20
Bilotta et al. [16]													
EBR_c_1.4x40_1	17.43	1.74	20	120	160	925	0.013	1.40	171	3.23	4.17	7.02	18.25
EBR_c_1.4x40_2	17.43	1.74	20	120	160	925	0.013	1.40	171	4.42	4.20	7.02	17.60
Fu et al. [17]													
B1S1	49	4.9	33	200	450	1300	0.008	0.67	251	286.39	83.90	61.75	137.70
B1S2	25.9	2.6	24	200	450	1300	0.005	0.67	251	149.99	50.20	15.82	121.20

## Data Availability

Data is contained within the article.
